# Supplementary material for: Adult stem cell activity in naked mole rats for long-term tissue maintenance
Source: Nat Commun. 2023 Dec 20;14:8484. doi: 10.1038/s41467-023-44138-6 (PMC10733326; doi:10.1038/s41467-023-44138-6)
Supplement: Supplementary file 1 — Supplementary Information file [file 41467_2023_44138_MOESM1_ESM.pdf]

Adult stem cell activity in naked mole rats for long-term tissue maintenance

SUPPLEMENTARY INFORMATION

SUPPLEMENTARY FIGURES

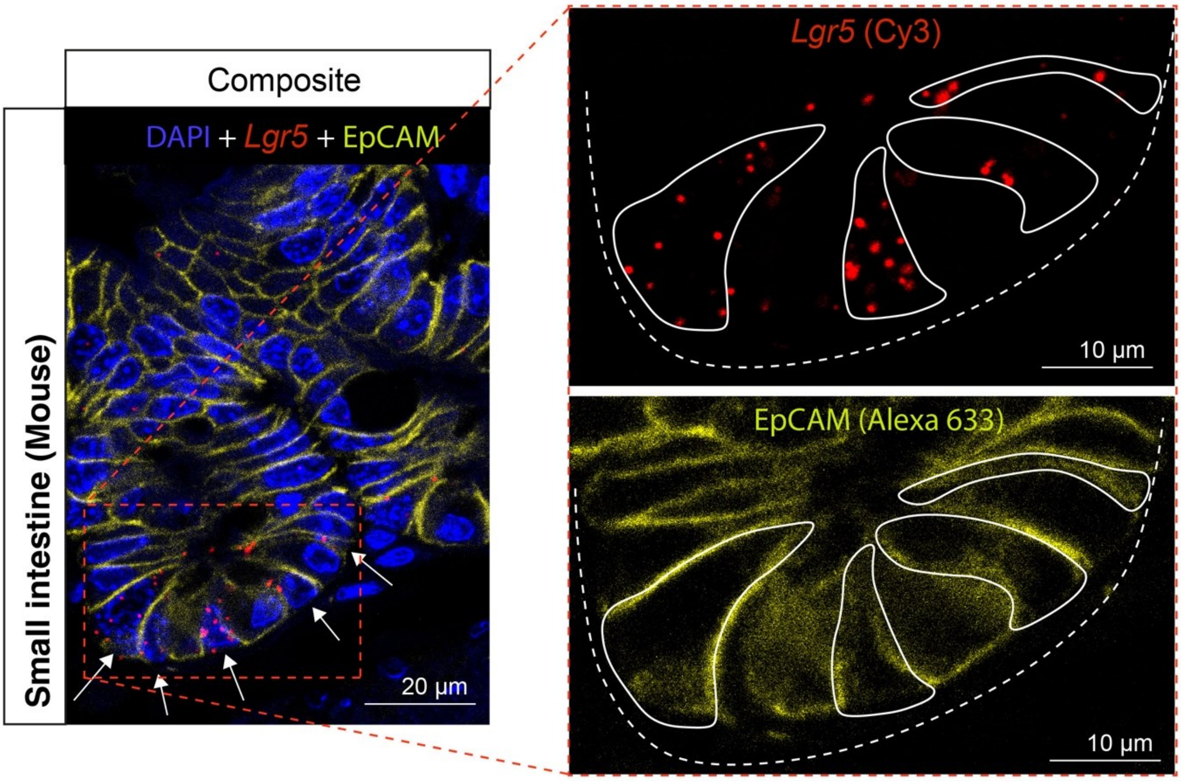

Supplementary Fig. 1: Representative composite and 2X digitally zoomed-in single-channel fluorescence images of mouse small intestinal crypts. Maximum-intensity *z*-projections of 4 µm thick image stacks of mouse tissue sections co-stained with *Lgr5* RNAscope probe (red), anti-EpCAM antibody (yellow) and DAPI (blue), White arrows indicate *Lgr5*-expressing (*Lgr5*$^{+}$) cells. The periphery of *Lgr5*$^{+}$ cells (containing >3 fluorescent puncta of mRNA transcripts) is outlined in white lines on each single channel image and is based on EpCAM staining. Scale bars are shown as 10 µm and 20 µm.

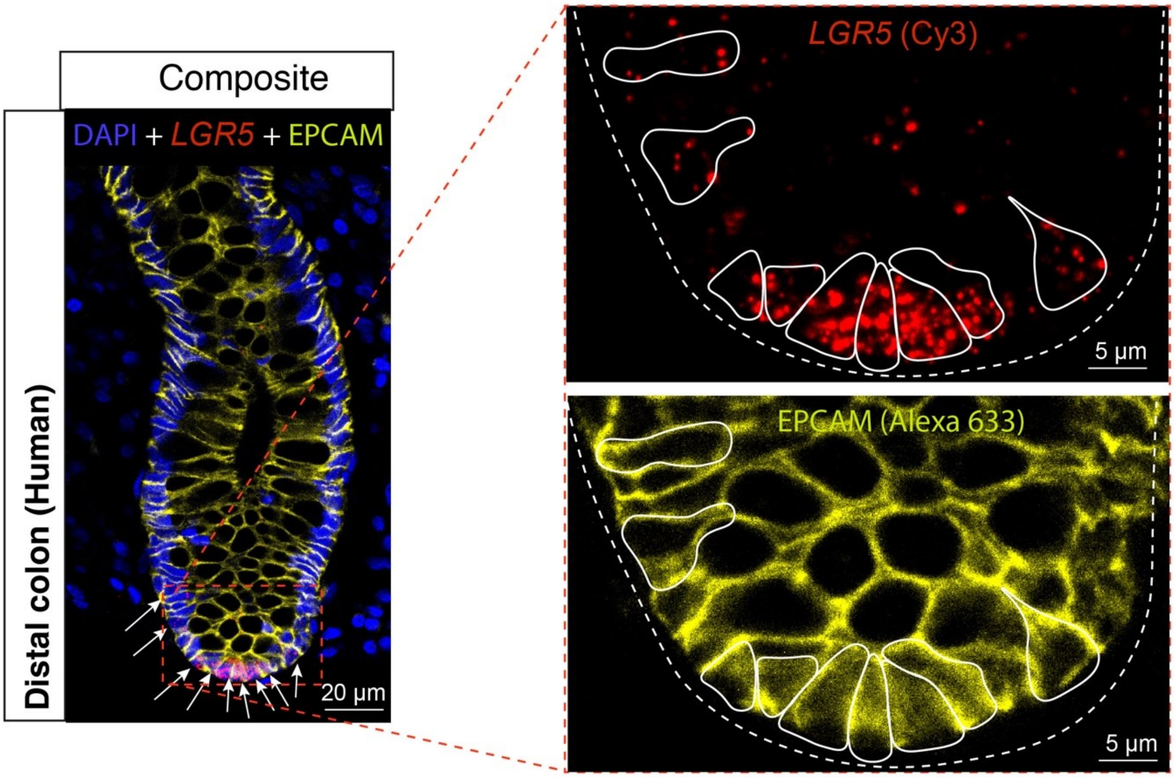

Supplementary Fig. 2: High resolution immunofluorescence images of human colonic crypts. Composite and 4X digitally zoomed-in single-channel confocal images of human colonic crypts co-stained with *LGR5* RNAscope probe (red), anti-EPCAM antibody (yellow) and DAPI (blue). White arrows show *LGR5*-expressing (*LGR5*$^{+}$) cells. Using EPCAM staining to identify individual cells, *LGR5*$^{+}$ cells have been outlined in white lines on each single-channel image. Scale bars are shown as 10 µm and 20 µm.

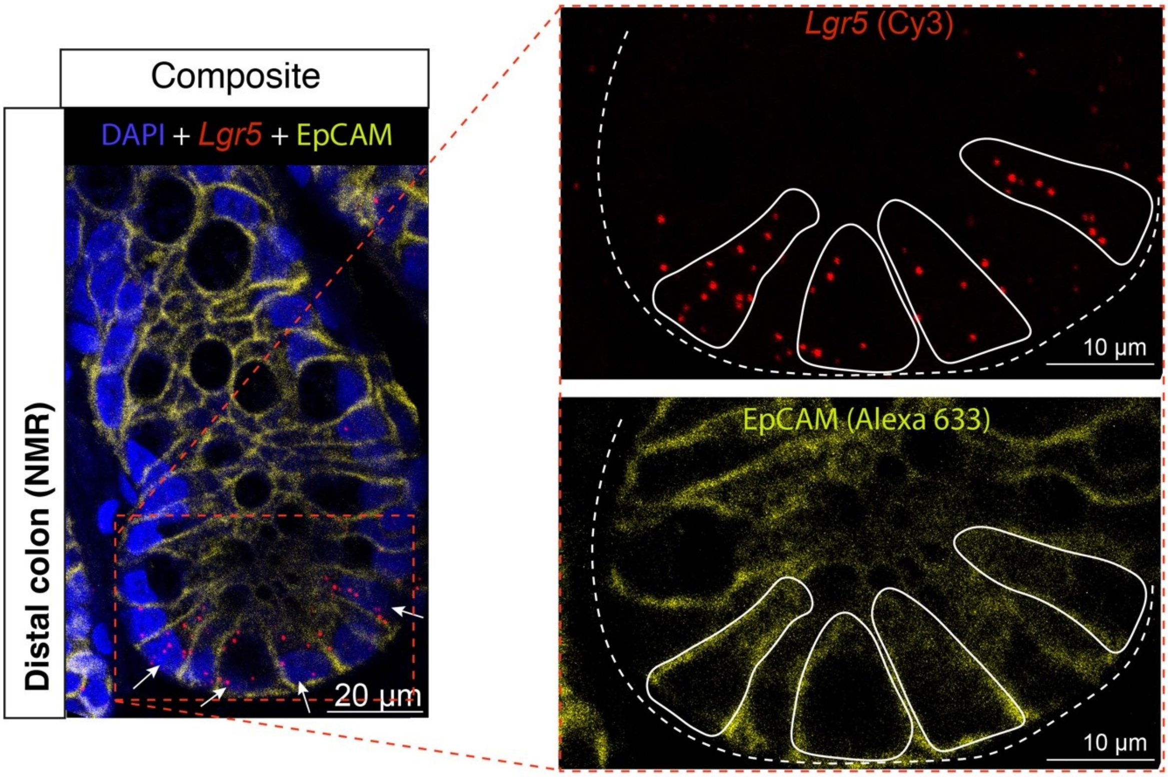

Supplementary Fig. 3: Immunofluorescence images of naked mole rat colonic crypts. Composite and 2X digitally zoomed single-channel confocal images of naked mole rat colonic tissues, co-stained with *Lgr5* RNAscope probe (red), anti-EpCAM antibody (yellow) and DAPI (blue). *Lgr5*-expressing (*Lgr5*$^{+}$) cells are shown by white arrows and white lines demarcate the periphery of *Lgr5*$^{+}$ cells on each single-channel image. Scale bars are shown as 10 µm and 20 µm.

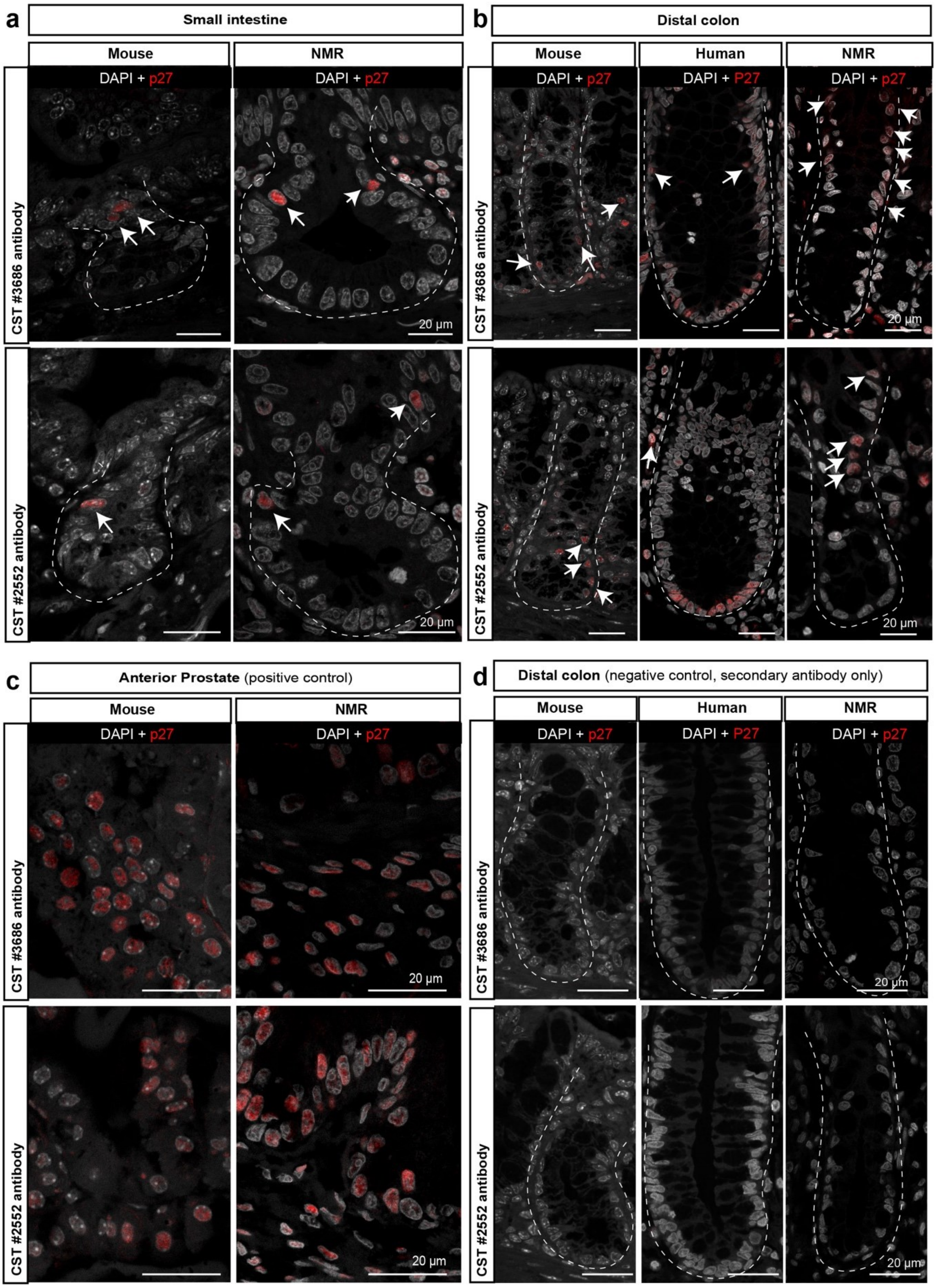

Supplementary Fig. 4: Expression of p27 or P27 in mouse, NMR and human intestine. Representative confocal images showing intestinal crypts stained with anti-

p27 or P27 antibodies (Cell Signaling Technology, 3686 or CST, 2552) (red) and counterstained with DAPI (grey) in **a**, the small intestine of wild-caught mouse (12-months-old) and wild-caught NMR (12-months-old) and **b**, in mouse (12-months-old), human (28 to 33-year-old) and NMR (12-months-old) colon. Dashed lines demarcate the outer periphery of intestinal crypts. White arrows indicate $p27^+$ or $P27^+$ cells above the crypt base to the top of the crypts. **c**, Immunofluorescent images of the mouse (12-months-old) and NMR (12-months-old) anterior prostate stained with anti-p27 antibodies (red) and DAPI (grey). **d**, Confocal images of crypts from mouse, human, and NMR distal colon stained with only secondary antibody (Invitrogen, #A21428) and counterstained with DAPI (grey). In **a-d**, the top panels show staining with CST #3686 antibody and the bottom panels show staining with CST #2552. All images in this figure were acquired using a 40X objective lens (1.1 W Corr) on a Zeiss 780 LSM upright confocal microscope. Scale bars are indicated on the images (20 μm).

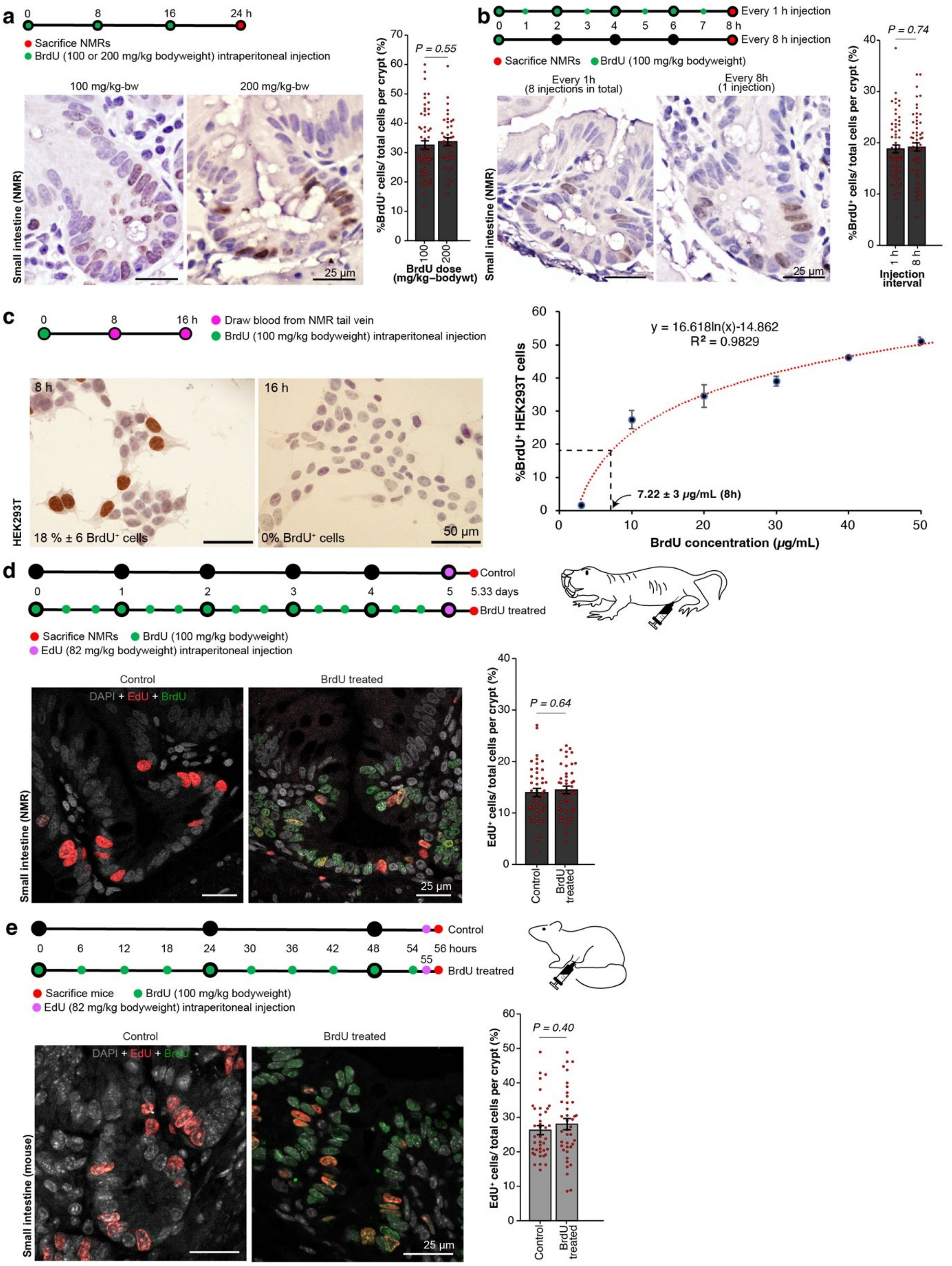

Supplementary Fig. 5 | Optimising experimental parameters for administering BrdU in naked mole rats and mice *in vivo.* a, Top, schema showing timeline of BrdU injections in NMRs (green) at two different doses and time of tissue harvest (red).

Bottom, Photomicrographs showing duodenal crypts of NMRs stained with anti-BrdU antibody (brown) and haematoxylin (blue). Bar graphs showing the mean percentage (±SEM) of $BrdU^+$ cells in the duodenal crypts of NMRs at two different doses of BrdU (*n*=42 crypts counted from 2 animals/dose). **b**, Top, schema illustrating two different frequencies of BrdU injections (green) and time of tissue collection (red). Bottom, Brightfield images showing duodenal crypts of NMRs stained with anti-BrdU antibody (brown) and haematoxylin (blue). Bar graphs showing the mean percentage (±SEM) of $BrdU^+$ cells in the duodenal crypt of NMRs injected at different frequencies (*n*=60 crypts from 2 animals /group). **c**, Top, schema showing blood collection times (red) from NMRs after one BrdU injection (green). Bottom, photomicrographs of HEK293T cells stained with anti-BrdU antibody (brown) and haematoxylin (blue). The mean percentages (±SEM) of $BrdU^+$HEK293T cells supplemented with sera extracted after 8 h (*n*=2 animals) and 16 h (*n*=2 animals) of BrdU injections are presented on the images. Right, A standard curve showing the mean percentage (±SEM) (*n*=3 wells) of $BrdU^+$HEK293T cells with increasing concentrations of BrdU. The concentration of BrdU in NMR sera after 8 h of BrdU injection (18% labelled cells) was derived from the graph (7.22 µg/ml ±3). **d-e**, Top, schema showing injection of 2'-deoxy-5-ethynyluridine (EdU) (purple) in **d**, NMRs and **e**, mice that had received no BrdU injection (control) or successive BrdU injections (green). Bottom, confocal images showing duodenal crypts of **d**, NMR and **e**, mouse stained with EdU-Alexa488 (red), anti-BrdU antibody (green) and DAPI (grey). Bar graphs showing the mean percentage (±SEM) of EdU^+ cells in duodenal crypts of **d**, NMRs and **e**, mice receiving no BrdU injection or multiple BrdU injections (*n*=40 crypts from 2 animals/group). *P*-values indicated on each graph were determined by Student's *t*-tests using two-tailed,

unpaired and unequal variance. Scale bars are indicated on the images (25 µm or 50 µm).

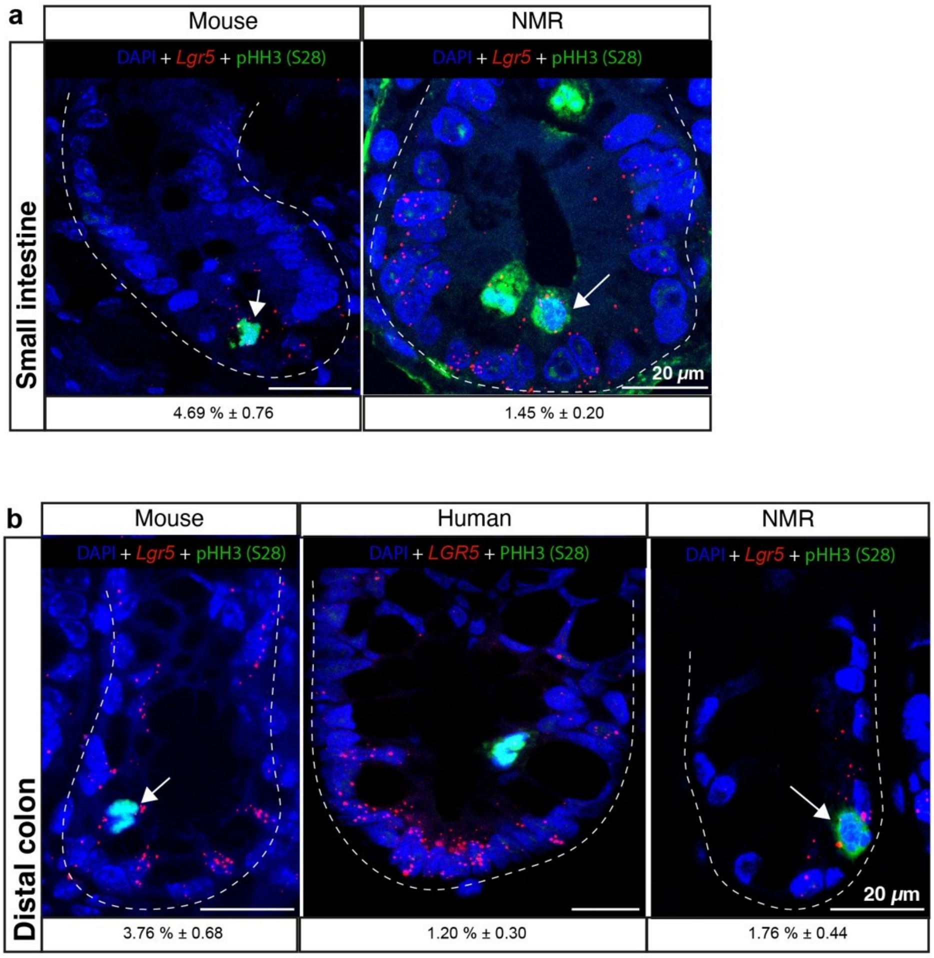

Supplementary Fig. 6: Mitotic index (phospho-histone-H3(Ser28)$^{+}$) of *Lgr5* or *LGR5*$^{+CBC}$ cells. Confocal images showing intestinal crypts stained with *Lgr5* or *LGR5*-mRNA ISH (red), anti-phospho-histone-H3(Ser28) antibody (green) and counterstained with DAPI (blue) in **a**, the small intestinal crypt of wild-caught mouse (12-months old) and wild-caught NMR (12-months old) and in **b**, colon of mouse, human (65 to 70 year-old) and NMR. Mean percentages (±SEM) of *Lgr5*$^{+CBC}$ or *LGR5*$^{+CBC}$ cells positive for PHH3(ser28) per crypt have been shown under each image (*n*=150 crypts counted from 3 individuals per species). Dashed lines demarcate

the outer periphery of each crypt. White arrows indicate *Lgr5*$^{+CBC}$ or *LGR5*$^{+CBC}$ cells positive for phospho-histone-H3(Ser28). Scale bars are indicated on the images (20 µm).

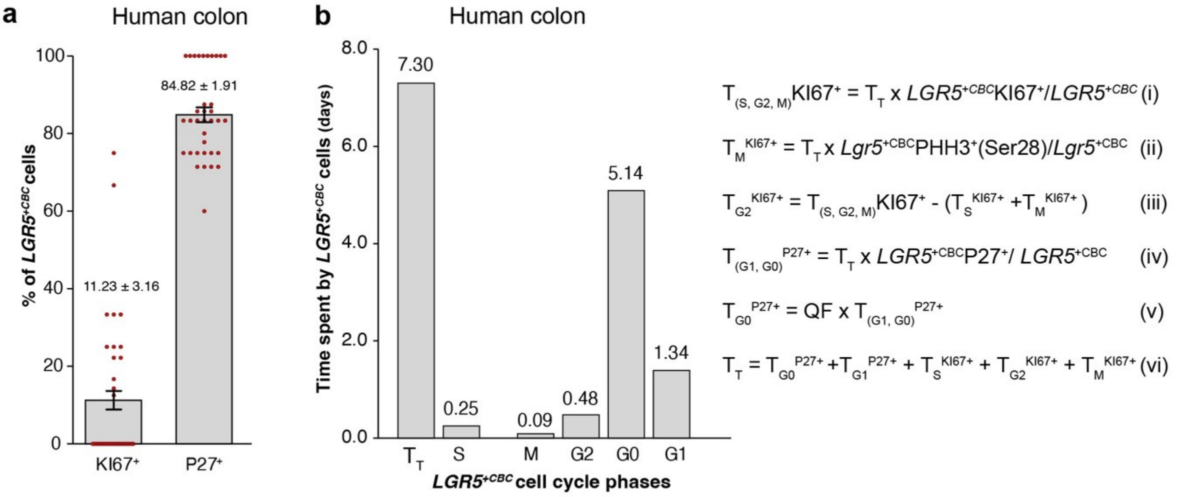

Supplementary Fig. 7: Duration of specific cell cycle phases in human colonic *LGR5*$^{+CBC}$ cells. a, Bar graph showing mean percentages (±SEM) of *LGR5*$^{+CBC}$ cells positive for KI67 or P27 expression in the human colon (*n*=35 crypts counted from 5 individuals aged between 65 to 70 years). **b**, Bar graph showing the duration of each phase of the cell cycle in the human colonic *LGR5*$^{+CBC}$ cells which have been shown to cycle every 7.3 days (T_T)[1], with the S phase of 0.25 days[1]. Using the fraction of *LGR5*$^{+CBC}$ cells positive for KI67, we calculated the time these cells take to transition from S to M phase using equation (i) shown alongside the graph above. The fraction of *LGR5*$^{+CBC}$ cells that scored positive for PHH3(S28) (1.2% ±0.30) was used to estimate the duration of M-phase (T_M) using equation (ii). The time spent in the G2 phase (T_{G2}) was calculated using equation (iii). We estimated the duration of G0 and G1 ($T_{G0, G1}$) in human colonic *LGR5*$^{+CBC}$ cells by using the proportion of *LGR5*$^{+CBC}$ P27^{+} and T_T in equation (iv). T_{G0} was estimated using the reported proportion of *LGR5*$^{+}$P27^{+}

cells in G0 (Quiescence Fraction, QF= 83%)[1] in equation (v). T_{G1} was estimated using equation (vi).

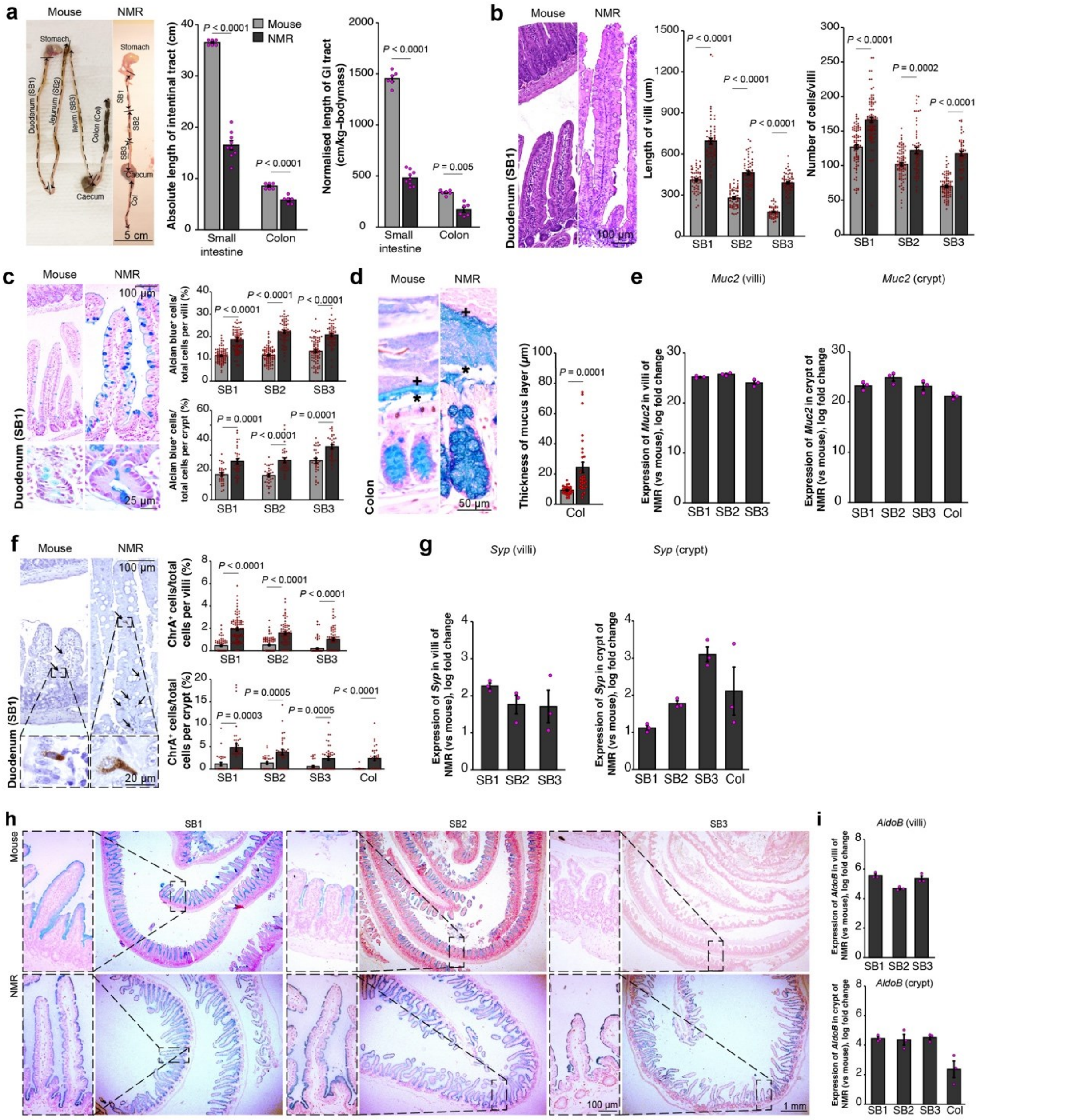

Supplementary Fig. 8: Macroscopic and microscopic analysis of wild-caught naked mole rat (NMR) versus wild-caught mouse intestine

a, Left, photographs showing the intestinal tracts of mice and NMRs. Right, bar graphs showing the mean length (±SEM) of mouse and NMR intestine (*n*=6 animals/group).

b, Left, H&E stained images of mouse and NMR duodenal villi. Right, bar graphs showing the mean (±SEM) of villus length and villus cell counts (*n*=60 villi from 6 animals/group). **c**, Left, microscopic images showing Alcian blue staining in mouse and NMR duodenum. Right, bar graphs showing the mean percentages (± SEM) of Alcian blue^{+} cells in the villi and the crypts of the duodenum (SB1), jejunum (SB2) and ileum (SB3) of mice and NMRs (*n*=60 villi counted from 6 animals/group; *n*=30 crypts from 3 animals/group). **d**, Left, brightfield microscopic images of Alcian blue staining in the mouse and NMR colon. The inner mucus layer is denoted by * and the outer layer by +. Right, bar graphs showing the mean thickness (±SEM) of the mucus layer in mouse and NMR colon (*n*=30 measurements taken from 3 animals/group). **e**, Bar graphs showing relative mean (±SEM) expression levels of *Muc2* in NMR villi (left) and crypts (right) compared to mice (*n*=3 animals/group) **f**, Left, microscopic images showing Chromogranin A staining in mouse and NMR duodenum. Right, bar graphs showing the percentage (±SEM) of Chromogranin A^{+} cells in the NMR villi and crypts of SB1, SB2, SB3 and colon compared to mice (*n*=60 villi from 6 animals/group; *n*=30 crypts from 3 animals/group). **g**, qRT-PCR measurement of *Synaptophysin* gene expression levels in NMR villi and crypts (mean ±SEM, *n*=3 animals/group). **h,** Photomicrographs of alkaline phosphatase staining in the small intestine of the mouse (top panel) and NMR (bottom panel). **i**, qRT-PCR analysis of *Aldolase B* expression in NMR villi (top) and crypts (bottom) compared to mice (mean ±SEM, *n*=3 animals/group). In all cases, Student's *t*-tests using two-tailed, unpaired and unequal variance were employed. Exact *P*-values are indicated on graphs. Scale bars are indicated on the images (20 µm, 25 µm, 50 µm, 100 µm, 1 mm, 5 cm).

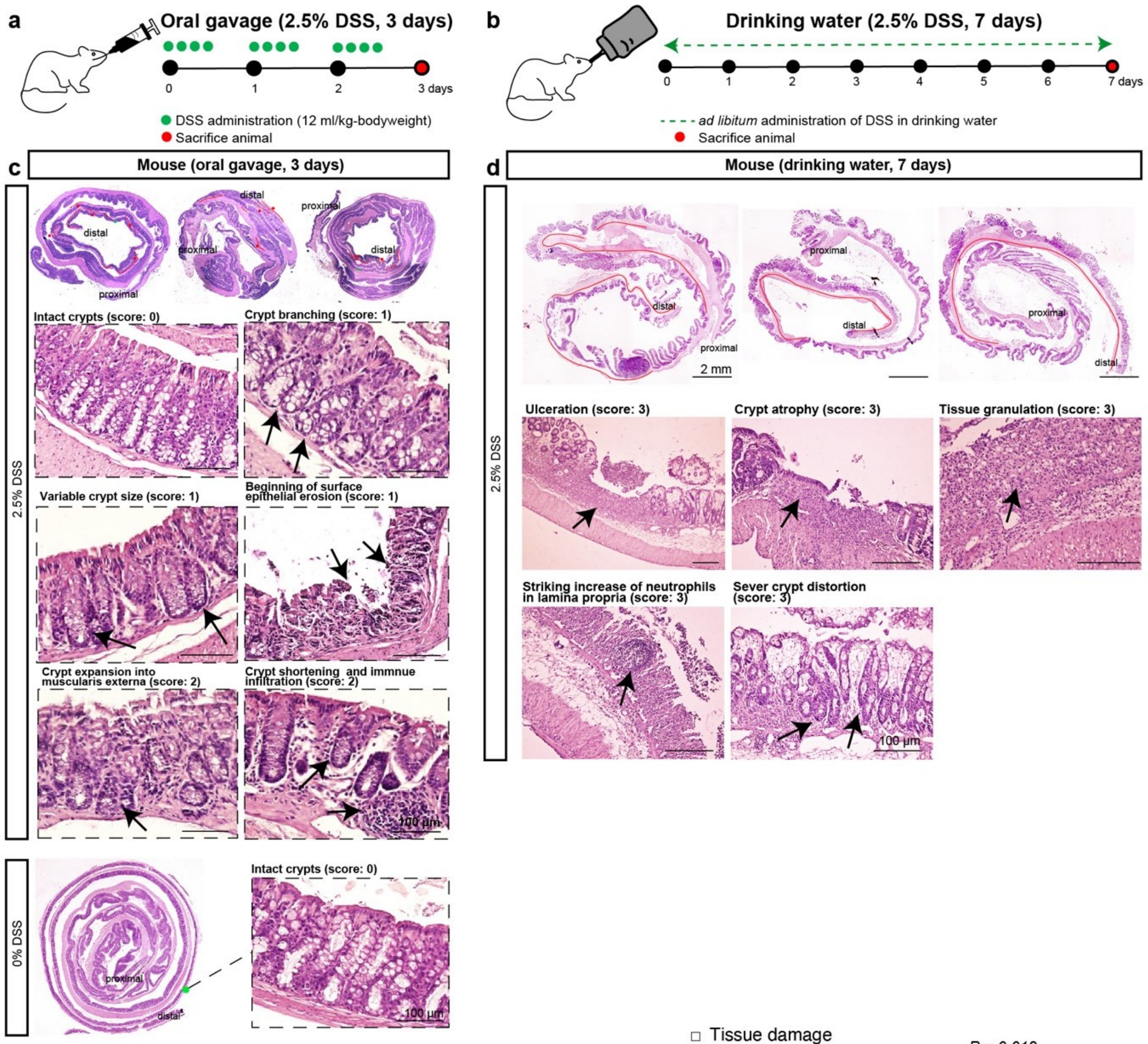

e

Scoring Matrix				
**Level**	**Score**	**Tissue Damage**	**Infiltration**	**Surface Involvement**
None	0	None	Infrequent	None
Mild	1	Isolated focal epithelial damage	Increased, some neutrophils	1-25%
Moderate	2	Mucosal erosions and ulceration	Submucosal present of inflammatory cell clusters	26-50%
Severe	3	Extensive damage deep into bowel wall	Transmural Cell Infiltration	51-75%
Extreme	4	--	--	76-100%

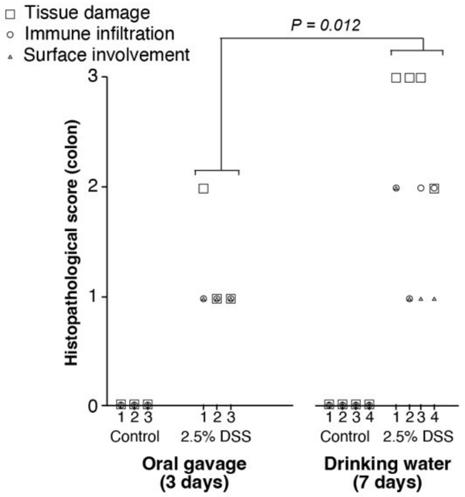

Supplementary Fig. 9: Comparison of intestinal damage in mice given dextran sodium sulphate (DSS) by two different methods

a, Schema illustrating the experimental design for administering 2.5% DSS via oral gavage at specific time points (green dots) for 3 days and day of tissue harvest (red dot) in C57BL/6J mice (2 to 4-months-old). **b**, Top, Schematic representation of the experimental strategy used to administer 2.5% DSS in drinking water for 7 days (green dashed line) and day of tissue harvest (red dot) in C57BL/6J mice (2 to 4-months-old). **c**, Haematoxylin and Eosin staining of gut rolls showing areas of tissue damage in the distal colon (highlighted in red) in mice treated with 2.5% DSS by oral gavage for 3 days. Magnified images showing histopathology of colonic tissue in DSS-treated mice. Score 0 indicates intact crypts in undamaged regions. Score 1 shows crypt branching, variable crypt size and the beginning of surface epithelial erosion. Score 2 highlights regions of crypt expansion into the muscularis externa, crypt shortening and immune infiltration in the submucosa. Black arrows indicate abnormalities/damage. Bottom, H&E staining showing tissue architecture in the distal colon of control (0% DSS) mice. **d**, Haematoxylin and Eosin (H&E) staining of colonic gut rolls of mice treated with 2.5% DSS via drinking water for 7 days. Areas of severe tissue damage in the mid to distal colon are highlighted in red. Magnified images show ulceration, crypt atrophy, tissue granulation, a striking increase in neutrophil infiltration and severe crypt distortion (Score 3). **e**, A table (left) showing the criteria used by pathologists to score tissue damage in the colon. Right, histopathological scoring of mice treated with 2.5% DSS for 3 days (n=3 animals) and mice treated for 7 days (n=4 animals). The P-value shown on the graph was determined by using a two-tailed Wilcoxon rank sum test. Numbers on the x-axis refer to individual animals in control and treated groups.

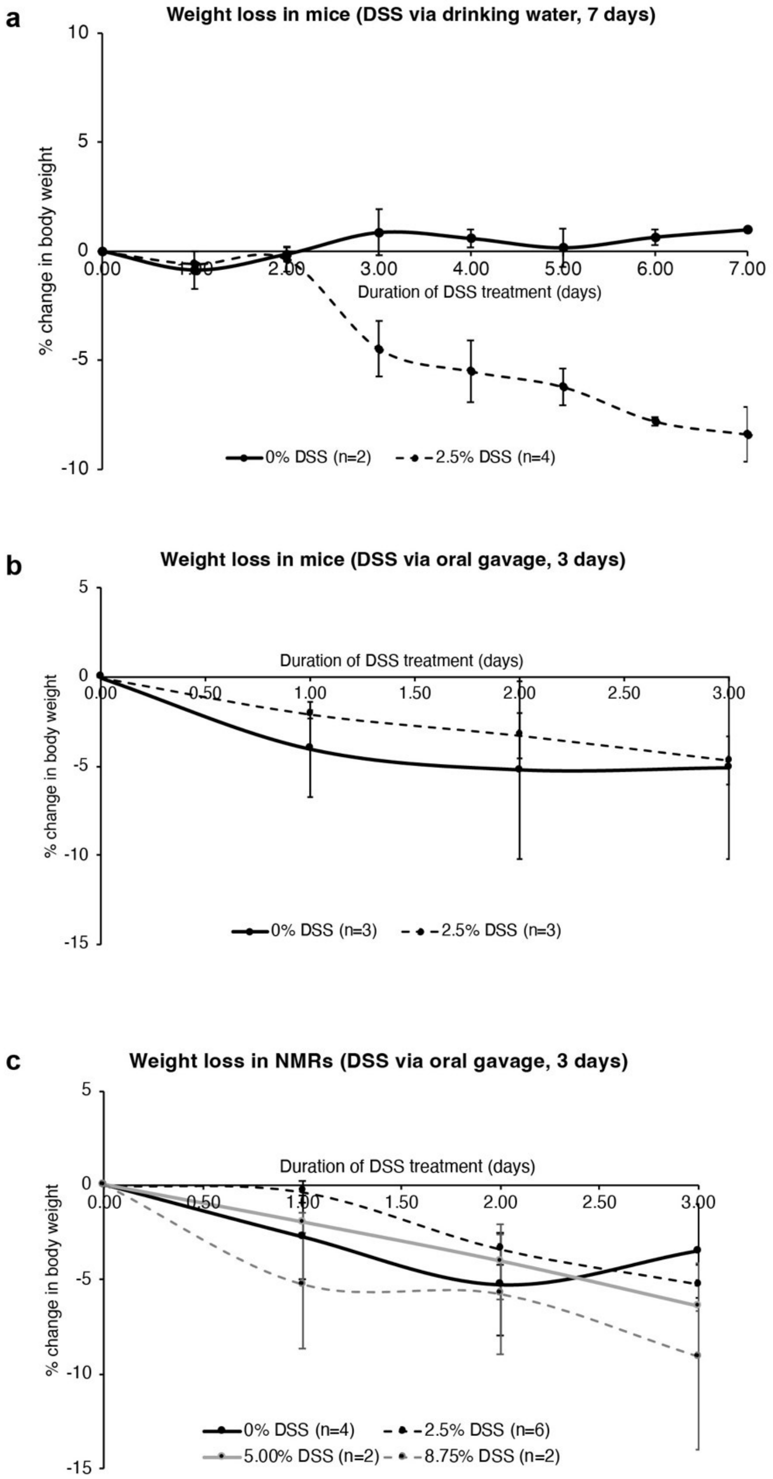

Supplementary Fig. 10: Change in body weight during dextran sodium sulphate (DSS) treatment in mice and NMRs. a, Change in the percentage of initial body weight with time in C57BL/6J mice receiving 0% (*n*=2 animals) or 2.5% DSS (*n*=4

animals) in water *ad libitum* for 7 days. **b**, Line graph showing gradual decline in the percentage of initial body weight over time in C57BL/6J mice treated with 0% or 2.5% DSS by oral gavaging at a frequency of four times per day for a total of 3 days (*n*=3 animals/group). **c**, Line graph displaying the change in the percentage of initial body weight with time in NMRs treated with 0% (*n*=4 animals), 2.5% (*n*=6 animals), 5.0% (*n*=2 animals), and 8.75% (*n*=2 animals) DSS by oral gavage (four times per day) for 3 days. Each data point represents the mean (± SEM).

a

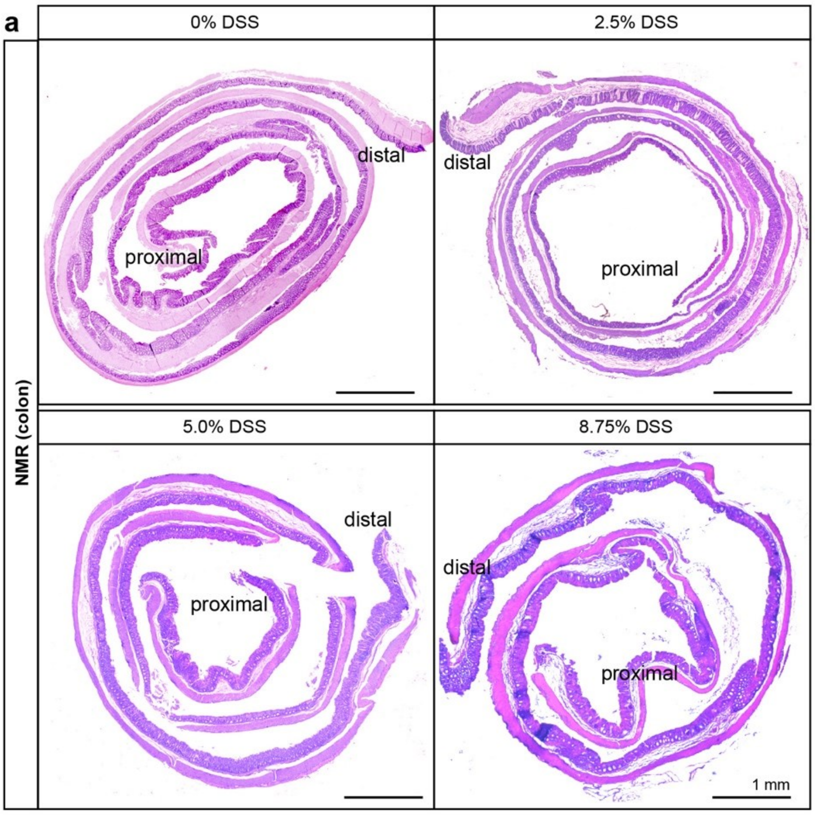

Scoring Matrix				
**Level**	**Score**	**Tissue Damage**	**Infiltration**	**Surface Involvement**
None	0	None	Infrequent	None
Mild	1	Isolated focal epithelial damage	Increased, some neutrophils	1-25%
Moderate	2	Mucosal erosions and ulceration	Submucosal present of inflammatory cell clusters	26-50%
Severe	3	Extensive damage deep into bowel wall	Transmural Cell Infiltration	51-75%
Extreme	4	--	--	76-100%

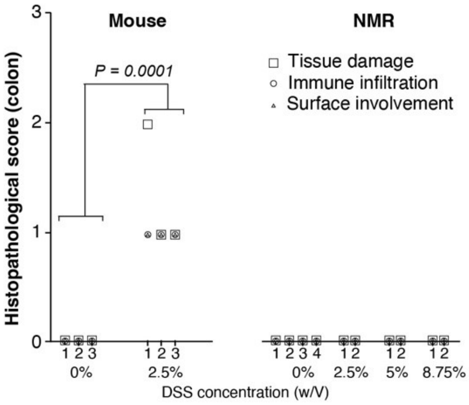

b

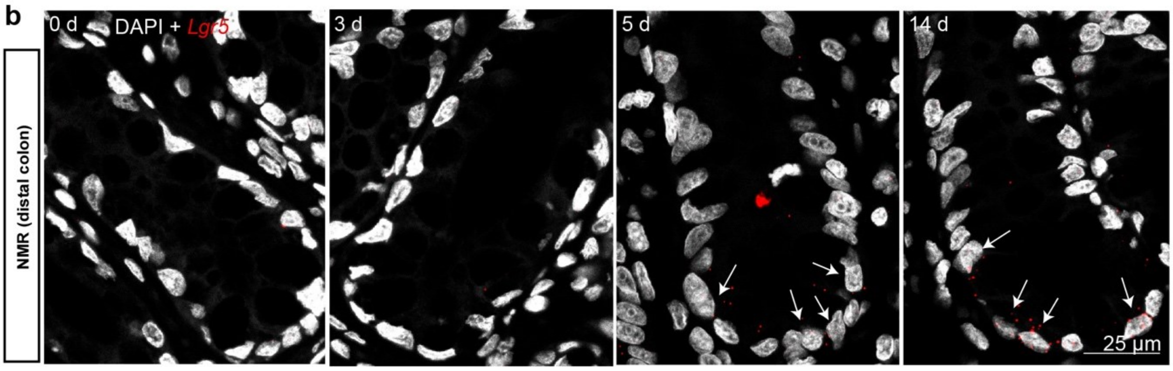

Supplementary Fig. 11: **Histological evaluation of NMR and mouse colon after treatment with DSS**

a, Top, Haematoxylin and Eosin staining of gut (colon) rolls from wild-caught NMRs (6 to 24-months-old) treated with 0%, 2.5%, 5.0% and 8.75% DSS via oral gavage for 3 days. Bottom left, table showing the histopathological criteria used to score tissue damage in the colon. Right, histopathological scoring of colons of C57BL/6J mice (2 to 4-months-old) treated with 0% and 2.5% DSS via oral gavage for 3 days (*n*=3 animals/group) and of NMRs treated with 0% (*n*=4 animals), 2.5% (*n*=2 animals), 5.0% (*n*=2 animals) and 8.75% (*n*=2 animals) DSS. The extent of tissue damage is shown by a square, immune infiltration by a circle and surface involvement by a triangle. The *P*-value shown on the graph was determined by using a two-tailed Wilcoxon rank sum test. Numbers on the x-axis refer to individual animals per group. **b**, Confocal images showing staining with NMR-specific RNAscope *Lgr5* probe (red) and DAPI (grey) in colonic crypts of NMRs after 0 days, 3 days, 5 days and 14 days of 2.5% DSS withdrawal. *Lgr5*$^+$ cells are marked by white arrows. Scale bars are indicated on the images (25 µm and 1 mm).

SUPPLEMENTARY TABLES

Supplementary Table 1. Summary of histological comparison between wild-caught NMR and wild-caught mouse intestine

	**Small intestine**			**Colon**		
	Mouse*	NMR*	*P*-value**	Mouse*	NMR*	*P*-value**
Length of GI track (cm)	36.5 ± 0.22	16.5 ± 0.88	<0.0001	8.5 ± 0.22	5.83 ± 0.31	<0.0001
Length of villi (μm)	288.01 ± 8.60	515.15 ± 13.13	<0.0001	---		
Number of cells/villi	100.23 ± 1.99	140.36 ± 2.72	<0.0001	---		
Alcian$^+$ cell/villi (%)	12.11 ± 0.26	20.23 ± 0.46	<0.0001	---		
Alcian$^+$ cell/crypt (%)	19.79 ± 0.88	29.27 ± 1.03	<0.0001	---		
Thickness of mucus layer (μm)	---			9.19 ± 0.48	24.37 ± 3.46	0.0001
ChrA$^+$ cell/villi (%)	0.38 ± 0.04	1.57 ± 0.08	<0.0001	---		
ChrA$^+$ cell/crypt (%)	1.00 ± 0.18	3.59 ± 0.38	0.0005	0.05 ± 0.05	2.38 ± 0.48	<0.0001
Lgr5$^+$ cell/crypt (%)	10.58 ± 0.55	26.62 ± 0.70	<0.0001	4.25 ± 0.37	6.64 ± 0.38	<0.0001

*mean ± standard error of mean

***P*-value. Student's *t*-tests using two-tailed, unpaired and unequal variance was employed.

Supplementary Table 2. Summary of changes observed in the intestinal crypts following 2.5% DSS treatment in NMRs and mice.

	**NMR (2.5% DSS for 3 days via oral gavage)**		**Mouse (2.5% DSS for 3 days via oral gavage)**		**Mouse (Davidson et al[2]) (2.5% DSS for 5 days via drinking water)**	
	**Distal colon**	**Duodenum**	**Distal colon**	**Duodenum**	**Distal colon**	**Proximal colon**
Number of *Lgr5*$^{+CBC}$ cells/crypt	100% loss	86% loss	No change	No change	100% loss	No change
Apoptotic index (Number of TUNEL^{+} cells/ 100 crypt) at crypt base	18-fold increase	4-fold increase	3-fold increase	3-fold increase	*not available*	*not available*
Apoptotic index (Number of TUNEL^{+} cells/ 100 crypt) above crypt base	3-fold increase	7-fold increase	2-fold increase	No change	*not available*	*not available*
Lgr5^{+CBC}Ki67^{+}/ *Lgr5*$^{+CBC}$ (%)	*not available*	No change	No change	No change	*not available*	*not available*
Ki67^{+} cells/total cells per crypt (%)	88% decrease	62% decrease	24% decrease	13% decrease	*not available*	*not available*
% of crypt with EdU+ cells	*not available*	*not available*	*not available*	*not available*	90% decrease	35% decrease
Recovery of *Lgr5*$^{+CBC}$	5 days	3 days	N/A	N/A	6 days	*not available*

% or fold difference has been calculated by comparing 2.5% DSS treated animals with controls.

SUPPLEMENTARY NOTES

Supplementary Note 1: Optimising experimental parameters for administering BrdU in naked mole rats and mice *in vivo*

We first determined the appropriate dose of BrdU to be administered in NMRs by injecting NMRs intraperitoneally with 100mg/kg and 200mg/kg bodyweight of BrdU, and found no difference in the percentage of cells labelled in the small intestine between the two doses although the staining with anti-BrdU antibody was stronger at the higher concentration (Supplementary Fig. 5a). As BrdU labelled cells were reliably detected at 100mg/kg, we used this concentration in subsequent experiments for reducing stress in animals as larger injection volumes (>350 μl) would be required for 200mg/kg given that BrdU solubility in aqueous solution is 50mM. As each injection of BrdU labels only those proliferating cells that are in the S-phase, it was important to design an appropriate timeline for injections to ensure maximal availability of BrdU in all proliferating cells of NMR crypts. We found no difference in the percentage of $BrdU^{+}$ cells per crypt section between animals injected every hour for 8 hours and those pulsed once and tissue assessed after 8 hours (Supplementary Fig. 5b). We next assessed the bioavailability of BrdU in NMRs over a specific period post-injection by withdrawing blood from animals 8 hours and 16 hours after a single injection at time 0 (Supplementary Fig. 5c, top). The plasma collected at these time points was added to the growth medium of HEK293T cells and BrdU uptake by these cells was detected by anti-BrdU immunolabelling (Supplementary Fig. 5c, left). We observed 18% ± 6 of BrdU-labelled HEK293T cells (labelling index) from plasma collected at 8 h while no BrdU-positive cells were detected in wells supplemented with NMR plasma collected at 16 h after a single BrdU injection (Supplementary Fig. 5c, left). We generated a standard curve by quantifying BrdU-labelled HEK293T cells at known concentrations

of BrdU that were added to the culture media (3, 10, 20, 30, 40, 50 μg/ml) (Supplementary Fig. 5c, right). Using this standard curve, we found the concentration of BrdU in NMR plasma after 8 h post-injection, when 18% ±6 of cells scored positive for BrdU, was 7.22 μg/ml ±3 (Supplementary Fig. 5c, right). Interestingly, a previous study using the same method in mouse, canary and quail has shown a faster clearance of BrdU (1 to 2 hours) in these species[3]. Given that the time between each injection had to be shorter than Ts to ensure that no proliferating cell passes completely through the S phase without being exposed to BrdU, and the period of BrdU injections had to be longer than Ts so that all proliferating cells pass through S at least once[4], we decided to use 8 h interval between each injection in NMRs and a total of 15 injections for 5 continuous days in our experimental design (Fig. 3a, 4a). Finally, to ensure that repeated injections of BrdU did not change the cell cycle duration by stressing the intestinal cells *in vivo*, we injected a second thymidine analogue, EdU, in NMRs that had received no BrdU injection (control) and those that had previously been subjected to 15 consecutive BrdU injections (BrdU treated) (Supplementary Fig. 5d, top). Both groups received one EdU injection 8 hours prior to the termination of the experiment (Supplementary Fig. 5d, top). Analysis of small intestinal crypts after 8h of EdU injection showed a similar proportion of EdU^+ cells per crypt in controls (14% ±0.8) and BrdU treated (14.5% (± 0.7) NMRs (Supplementary Fig. 5d). We concluded that showing that the proportion of proliferating cells in the S-phase was unaffected by repeated BrdU injections.

We have also assessed the impact of repeated BrdU injections on the cellular kinetics of mouse small intestinal crypts by injecting EdU in mice that had received no BrdU injection (control) and mice that had received 10 rounds of BrdU injections (BrdU

treated) (Supplementary Fig. 5e, top). After one hour of EdU administration, we detected a similar percentage of EdU^+ cells in the duodenal crypts of both the control and BrdU-treated mice (Supplementary Fig. 5e). This indicated that repeated administration of BrdU in mice also did not impact the cellular kinetics in the intestinal crypts.

Supplementary Note 2: Naked mole rat intestinal epithelium has an expanded differentiation zone

Starting by characterising the macroscopic features of the intestine, we found that NMR small intestine and colon were significantly shorter in absolute length (55% and 31%, respectively) than the mouse (Supplementary Fig. 8a, left and Supplementary Table 1). Similarly, after normalising to body mass, NMR intestinal length was significantly shorter than mouse (Supplementary Fig. 8a, right). Histological analysis of these tissues showed that the intestinal villi in NMRs were approximately 79% longer than in mice, consistent with higher cell counts detected (Supplementary Fig. 8b).

We quantified any differential distribution of secretory (goblet and enteroendocrine) and absorptive (enterocytes) cell types between the two species. We observed a significantly higher proportion of goblet cells, assessed by Alcian blue staining, in both the crypts (48%) and villi (67%) of the NMR small intestine compared to mice (Supplementary Fig. 8c, Supplementary Table 1). In the colon due to the narrower structure of the colonic crypts in both species, it was not possible to accurately discern individual cells for counting after Alcian blue staining in fixed tissues (Supplementary Fig. 8d, left). We, therefore, measured the thickness of the surface mucus layer by

processing the colonic tissues in Carnoy's solution that stabilised the mucus gel before proceeding to stain the tissue with Alcian blue[5]. Measuring the distance from the inner to the outermost layer of the mucus gel lining the surface epithelium, we found that the NMR mucin layer was 1.65 times thicker compared to the mouse (Supplementary Fig. 8d right, Supplementary Table 1). Moreover, we also found higher gene expression levels of *Muc2*, a gene encoding the predominant gel-forming mucin in the intestine, in all regions of the NMR intestine compared to mice (Supplementary Fig. 8e).

Chromogranin A immunohistochemistry showed a higher proportion of enteroendocrine cells in both the NMR small intestine and colon compared to mice (Supplementary Fig. 8f, Supplementary Table 1). Real-time PCR also detected elevated levels of *Synaptophysin*, a gene expressed by synaptic-like microvesicles secreted by enteroendocrine cells, in NMR intestinal villi and crypts compared to murine counterparts (Supplementary Fig. 8g).

Using a histochemical assay to detect alkaline phosphatase (AP) activity in the absorptive enterocytes of the small intestine, we observed a gradual decrease in AP staining in the mouse villi from the proximal to distal regions (Supplementary Fig. 8h, top panel). Conversely, we observed a sustained expression of AP throughout the small intestine in NMR villi (Supplementary Fig. 8h, bottom panel). Quantitative-PCR analysis of the small intestinal crypts and villi revealed much higher expression of *AldolaseB,* another marker of enterocytes[6,7], in NMRs compared to mice (Supplementary Fig. 8i). In summary, both the NMR small intestine and colon possessed higher proportion of every differentiated cell type we assessed compared

to mouse intestine (Supplementary Table 1). We did not extend our analysis to Paneth, tuft or M cells.

Supplementary Note 3. Comparison of the intestinal damage induced in mice given dextran sodium sulphate (DSS) via oral gavage or in drinking water

A typical protocol for inducing intestinal injury in mice is to administer DSS in drinking water and damage is seen within 3-7 days[8,9]. In our study which included NMRs that do not drink water normally, we had to establish a new method that involved delivering DSS to animals via oral gavaging. As oral gavaging is an intrusive technique, we terminated the experiment after 3 days to minimise stress on animals. In a side-by-side comparison, we gavaged C57BL/6J mice (2 to 4-months-old, *n*=3) orally with 2.5% DSS for 3 days (Supplementary Fig. 9a) and supplemented the drinking water of another group of C57BL/6J mice (*n*=4) with 2.5% DSS for 7 days (Supplementary Fig. 9b). We next assessed the extent of intestinal damage in mice using these two methods of DSS delivery. We observed a mild to a modest degree of epithelial damage in 30% of the distal colon in mice administered with DSS via oral gavaging (Supplementary Fig. 9c). The damage to the colonic tissue architecture was characterized by branching and shortening of crypts, variation in crypt width, expansion of crypts into muscularis mucosa, and beginning of erosion of surface epithelium (Supplementary Fig. 9c). We also detected some immune cell clusters in these mice (Supplementary Fig. 9c). No crypt atrophy, tissue granulation, ulceration, or immune infiltration deep into bowel wall was present in these mice.

We then analysed the colons of mice administered with 2.5% DSS in drinking water for 7 days and detected severe epithelial damage in 80% of mid to distal colon

(Supplementary Fig. 9d) similar to previously published results[2]. Nearly 60% of distal colonic crypts in the affected areas underwent total crypt atrophy marked by flattening of mucosa, absence of crypts. We also observed necrotic debris above the ulcerated mucosa. The remaining 40% of the crypts exhibited severe crypt distortion such as glandular rarefaction, shortening and extensive branching of crypts, and there was a striking increase of neutrophils in lamina propria (Supplementary Fig. 9d). Two independent pathologists summarized their scoring of injuries in the two DSS models (Supplementary Fig. 9e). We concluded that oral gavaging for 3 days only caused mild to moderate intestinal injury which was similar to a chronic DSS model where mice were administered 1% DSS in drinking water over 42 days in 3 cycles of weekly administration and recovery[10].

Supplementary Note 4. Change in body weight during dextran sodium sulphate (DSS) treatment in mice and naked mole rats

In agreement with previous findings[11,12], we observed an 8.4% loss of initial body weight in mice receiving 2.5% DSS in drinking water by day 7, while the weight of control mice receiving unsupplemented water remained unchanged (Supplementary Fig. 10a). For mice treated with 0% or 2.5% DSS by 4 oral gavages per day (12 rounds of gavage in 3 days), we observed nearly 5% loss of initial body weight in both control and DSS-treated cohort by day 3 (Supplementary Fig. 10b). Similarly, we also observed nearly 5-9% loss of initial body weight in NMRs that received 0% or 2.5%, 5%, 8.5% DSS via oral gavage (Supplementary Fig. 10c). Therefore, weight loss in oral gavaged mice was not a measure of the effect of DSS, but was more reflective of the stress induced on these animals by oral gavaging itself. This supports our reasoning for conducting the experiment for only 3 days and not extending it further.

SUPPLEMENTARY REFERENCES

1 Ishikawa, K. *et al.* Identification of Quiescent LGR5(+) Stem Cells in the Human Colon. *Gastroenterology* (2022). https://doi.org:10.1053/j.gastro.2022.07.081

2 Davidson, L. A. *et al.* Alteration of colonic stem cell gene signatures during the regenerative response to injury. *Biochim Biophys Acta* **1822**, 1600-1607 (2012). https://doi.org:10.1016/j.bbadis.2012.06.011

3 Barker, J. M., Charlier, T. D., Ball, G. F. & Balthazart, J. A new method for in vitro detection of bromodeoxyuridine in serum: a proof of concept in a songbird species, the canary. *PLoS One* **8**, e63692 (2013). https://doi.org:10.1371/journal.pone.0063692

4 Nowakowski, R. S., Lewin, S. B. & Miller, M. W. Bromodeoxyuridine immunohistochemical determination of the lengths of the cell cycle and the DNA-synthetic phase for an anatomically defined population. *J Neurocytol* **18**, 311-318 (1989). https://doi.org:10.1007/BF01190834

5 Matsuo, K., Ota, H., Akamatsu, T., Sugiyama, A. & Katsuyama, T. Histochemistry of the surface mucous gel layer of the human colon. *Gut* **40**, 782-789 (1997). https://doi.org:10.1136/gut.40.6.782

6 Lukonin, I. *et al.* Phenotypic landscape of intestinal organoid regeneration. *Nature* **586**, 275-280 (2020). https://doi.org:10.1038/s41586-020-2776-9

7 Chang, J. *et al.* Proteomic changes during intestinal cell maturation in vivo. *J Proteomics* **71**, 530-546 (2008). https://doi.org:10.1016/j.jprot.2008.08.003

8 Okayasu, I. *et al.* A novel method in the induction of reliable experimental acute and chronic ulcerative colitis in mice. *Gastroenterology* **98**, 694-702 (1990). https://doi.org:10.1016/0016-5085(90)90290-h

9 Yan, Y. *et al.* Temporal and spatial analysis of clinical and molecular parameters in dextran sodium sulfate induced colitis. *PLoS One* **4**, e6073 (2009). https://doi.org:10.1371/journal.pone.0006073

10 Randall-Demllo, S. *et al.* Characterisation of colonic dysplasia-like epithelial atypia in murine colitis. *World J Gastroenterol* **22**, 8334-8348 (2016). https://doi.org:10.3748/wjg.v22.i37.8334

11 Bonfiglio, R. *et al.* Extensive Histopathological Characterization of Inflamed Bowel in the Dextran Sulfate Sodium Mouse Model with Emphasis on Clinically Relevant Biomarkers and Targets for Drug Development. *Int J Mol Sci* **22** (2021). https://doi.org:10.3390/ijms22042028

12 Araki, Y., Mukaisyo, K., Sugihara, H., Fujiyama, Y. & Hattori, T. Increased apoptosis and decreased proliferation of colonic epithelium in dextran sulfate sodium-induced colitis in mice. *Oncol Rep* **24**, 869-874 (2010). https://doi.org:10.3892/or.2010.869
