# Supplementary material for: Adult stem cell activity in naked mole rats for long-term tissue maintenance
Source: Nat Commun. 2023 Dec 20;14:8484. doi: 10.1038/s41467-023-44138-6 (PMC10733326; doi:10.1038/s41467-023-44138-6)
Supplement: Supplementary file 3 — Reporting Summary [file 41467_2023_44138_MOESM3_ESM.pdf]

nature portfolio

Corresponding author(s): Shazia Irshad, Ian Tomlinson

Last updated by author(s): Nov 19, 2023

Reporting Summary

Nature Portfolio wishes to improve the reproducibility of the work that we publish. This form provides structure for consistency and transparency in reporting. For further information on Nature Portfolio policies, see our Editorial Policies and the Editorial Policy Checklist.

Statistics

For all statistical analyses, confirm that the following items are present in the figure legend, table legend, main text, or Methods section.

n/a	Confirmed	
☐	☒	The exact sample size (n) for each experimental group/condition, given as a discrete number and unit of measurement
☐	☒	A statement on whether measurements were taken from distinct samples or whether the same sample was measured repeatedly
☐	☒	The statistical test(s) used AND whether they are one- or two-sided<br>*Only common tests should be described solely by name; describe more complex techniques in the Methods section.*
☒	☐	A description of all covariates tested
☒	☐	A description of any assumptions or corrections, such as tests of normality and adjustment for multiple comparisons
☐	☒	A full description of the statistical parameters including central tendency (e.g. means) or other basic estimates (e.g. regression coefficient) AND variation (e.g. standard deviation) or associated estimates of uncertainty (e.g. confidence intervals)
☐	☒	For null hypothesis testing, the test statistic (e.g. F, t, r) with confidence intervals, effect sizes, degrees of freedom and P value noted<br>*Give P values as exact values whenever suitable.*
☒	☐	For Bayesian analysis, information on the choice of priors and Markov chain Monte Carlo settings
☒	☐	For hierarchical and complex designs, identification of the appropriate level for tests and full reporting of outcomes
☒	☐	Estimates of effect sizes (e.g. Cohen's d, Pearson's r), indicating how they were calculated

Our web collection on statistics for biologists contains articles on many of the points above.

Software and code

Policy information about availability of computer code

Data collection	We have used Microsoft Excel (v16.77.1) for inputing raw data after collection.
Data analysis	We performed all the analysis and generated the graphs using STATA/MP 14.1 (StataCorp, TX, USA).

For manuscripts utilizing custom algorithms or software that are central to the research but not yet described in published literature, software must be made available to editors and reviewers. We strongly encourage code deposition in a community repository (e.g. GitHub). See the Nature Portfolio guidelines for submitting code & software for further information.

Data

Policy information about availability of data

All manuscripts must include a data availability statement. This statement should provide the following information, where applicable:

- Accession codes, unique identifiers, or web links for publicly available datasets
- A description of any restrictions on data availability
- For clinical datasets or third party data, please ensure that the statement adheres to our policy

Source data are provided as a "Source Data" file.

Human research participants

Policy information about studies involving human research participants and Sex and Gender in Research.

Reporting on sex and gender	We collected colonoscopy samples from both male and female individuals in each cohort of this study.
Population characteristics	We collected biopsy samples from healthy individuals belonging to three age cohorts: 28-33 y, 65-70 y and 71-74 y.
Recruitment	We obtained written informed consent from all participants undergoing routine bowel cancer or IBD screening. All samples were anonymized. Only healthy individuals with no sign of IBD/cancer confirmed by colonoscopy were included in this study.
Ethics oversight	We collected normal human colonoscopy samples under the research tissue bank ethics 16/YH/0247 supported by NIHR Biomedical Research Centre, Oxford, U.K. and under the London Dulwich Research Ethics Committee (reference number 15/LO/1998).

Note that full information on the approval of the study protocol must also be provided in the manuscript.

Field-specific reporting

Please select the one below that is the best fit for your research. If you are not sure, read the appropriate sections before making your selection.

☒ Life sciences ☐ Behavioural & social sciences ☐ Ecological, evolutionary & environmental sciences

For a reference copy of the document with all sections, see nature.com/documents/nr-reporting-summary-flat.pdf

Life sciences study design

All studies must disclose on these points even when the disclosure is negative.

Sample size	We did not perform any a priori sample size calculation.
Data exclusions	We excluded any mis-shaped crypt/villi due to slanted plane of cut in micro tome from our analysis. Only crypts/villi containing a single continuous layer of epithelial cells were included in the analysis.
Replication	For experiments involving DSS and BrdU, we performed the experiments on two different age groups (Fig. 3, 4, 6, 7) on two different years and reached similar conclusions from both datasets.
Randomization	We did not perform any randomization.
Blinding	We did not perform any blinding.

Reporting for specific materials, systems and methods

We require information from authors about some types of materials, experimental systems and methods used in many studies. Here, indicate whether each material, system or method listed is relevant to your study. If you are not sure if a list item applies to your research, read the appropriate section before selecting a response.

Materials & experimental systems

n/a	Involved in the study	
☐	☒	Antibodies
☐	☒	Eukaryotic cell lines
☒	☐	Palaeontology and archaeology
☐	☒	Animals and other organisms
☒	☐	Clinical data
☒	☐	Dual use research of concern

Methods

n/a	Involved in the study	
☒	☐	ChIP-seq
☒	☐	Flow cytometry
☒	☐	MRI-based neuroimaging

Antibodies

Antibodies used	Primary antibodies: Chromogranin A (AbCam, ab15160, lot#GR3229573-3), BrdU (AbCam, ab6326, lot#GR3289291-1), Ki67 (Cell Signaling, 12202, clone: D3B5, lot#5), EpCAM (Abcam, ab71916, lot#GR3266477-1), P27 (Cell SIgnalling, 3686, clone: D69C12, lot#4

	and Cell Signalling, 2552, lot#8) and PHH3 (AbCam, ab32388, lot#GR3273229-2)<br><br>Secondary antibodies: Goat anti-Rabbit IgG (H+L) Cross-Adsorbed Secondary Antibody, Alexa Fluor™ 488 (Invitrogen, A11008, lot#2743033), Goat anti-Rat IgG (H+L) Cross-Adsorbed Secondary Antibody, Alexa Fluor™ 488 (Invitrogen, A11006, lot#2480078), Goat anti-Rabbit IgG (H+L) Cross-Adsorbed Secondary Antibody, Alexa Fluor™ 555 (Invitrogen, A21428, lot#2308257), Goat anti-Rabbit IgG (H+L) Cross-Adsorbed Secondary Antibody, Alexa Fluor™ 633 (Invitrogen, A21070, lot#2306816), Goat Anti-Rat IgG H&L (Biotin) (Abcam, ab207997, lot#GR3262739-3)
Validation	For mouse and human tissues, these antibodies have been validated for immunohistochemistry/immunofluorescence by the commercial providers. A list of publications where each of these antibodies have been used in mouse/human tissues is available on the manufacturer's website.<br>For naked mole rat tissues, we validated the specificity of the antibodies to the target proteins by performing optimisation using different concentrations including negative controls (staining with secondary antibody only). While optimising each antibody in naked mole rat tissues, we also included mouse/human tissues (positive control) for which the antibody is validated by the commercial provider. Antibodies that only produced a strong specific signal in the target cellular compartment were chosen for our study.

Eukaryotic cell lines

Policy information about cell lines and Sex and Gender in Research

Cell line source(s)	HEK293T (ATCC, Catalogue: CRL-3216)
Authentication	We did not perform any authentication of the cell line.
Mycoplasma contamination	We tested the culture for mycoplasma using MycoAlert Kit (Lonza) and detected no contamination.
Commonly misidentified lines (See ICLAC register)	Not applicable

Animals and other research organisms

Policy information about studies involving animals; ARRIVE guidelines recommended for reporting animal research, and Sex and Gender in Research

Laboratory animals	We purchased C57BL/6J mice (2 to 4 month-old) used in this study from Charles River (Kent, UK) or the Jackson Laboratory (USA) and housed them at Biomedical Services Unit in John Radcliffe Hospital, Oxford, UK or at Rutgers University Animal Facility in Newark, New Jersey, USA. The animal facility was maintained at 19-23°C temperature, 45-65% relative humidity, and alternating 12h light/12h dark (7am to 7pm) cycle.
Wild animals	We acquired wild-caught mice (2-month- to 18-month-old) from a founder population which was trapped in lower Austria, Vienna (2016), transported by road in individually ventilated cages, and housed at the Konrad Lorenz Institute of Ethology, University of Vienna, Austria. All mice used in this study were sacrificed by cervical dislocation and intestinal tissues were harvested for histological analysis.<br><br>The wild-caught naked mole rats (6-month- to 36-month-old) used in this study are descended from multiple colonies captured by Prof. Jenny Jarvis, primarily in Mtito Andei and Lerata, Kenya, and constitute a mixed parentage (Jarvis 1981). The descendants of the founder population were collected from the animal facility of University of Cape Town (Jarvis lab), South Africa and transported in individually ventilated cages by road to the animal facility of the Department of Zoology and Entomology, University of Pretoria, South Africa. All naked mole rats used in this study were sacrificed by cervical dislocation to collect intestinal tissues for histological analysis.<br><br>Reference: J. U. Jarvis, Eusociality in a mammal: Cooperative breeding in naked mole-rat colonies. Science 212, 571–573 (1981). doi:10.1126/science.7209555
Reporting on sex	We included both male and female animals in each cohort of this study.
Field-collected samples	We housed the mice in individually ventilated cages under specific pathogen-free conditions, maintained at 19-23°C temperature, 45-65% relative humidity, in an alternating 12-hour light/12-hour dark (7am to 7pm) cycles, and fed with food and water ad libitum.<br><br>We kept the naked mole rats in tunnel systems consisting of several Perspex chambers containing wood shavings as nestling material. The NMR room was maintained at temperatures ranging between 29–32°C, with relative humidity around 40-60%. NMRs were fed chopped fresh fruits and vegetables (apple, sweet potato, cucumber, and capsicum) daily ad libitum along with weekly supplement of ProNutro (Bokomo). Since NMRs obtain all their necessary water from food sources, we did not provide additional drinking water to the animals.<br><br>All mice and naked mole rats used in this study were sacrificed by cervical dislocation to collect intestinal tissues for histological evaluation.
Ethics oversight	In this study, we performed animal procedures in four different countries: U.K, U.S.A, Austria, and the Republic of South Africa. We carried out all procedures in accordance with Home Office, UK regulations and the Animals (Scientific Procedures) Act, 1986 of UK, the Institutional Animal Care and Use Committee (IACUC) of USA, Act 7, 1991 of South Africa, and the Directive 2010/63/EU of the European Parliament.

We performed scientific procedures on naked mole rats under ethics approval (NAS046-19 and NAS289-2020) by the Animal Ethics Committee, University of Pretoria, South Africa.

Note that full information on the approval of the study protocol must also be provided in the manuscript.
